# QuEChERS-超高效液相色谱-串联质谱法测定马尿中33种非甾体抗炎药残留

**DOI:** 10.3724/SP.J.1123.2025.08014

**Published:** 2026-09-08

**Authors:** Xiaonan ZHANG, Zhichao MA, An LIU, Yuan HUANG, Xuehong MIN, Mei LIU, Zhengqi XIE, Yansong WEI

**Affiliations:** 1. 武汉商学院体育学院·国际马术学院，湖北 武汉 430056; 1. School of Physical Education and National Equestrian Academy，Wuhan Business University，Wuhan 430056，China; 2. 运动马检测及应用转化湖北省工程研究中心，湖北 武汉 430056; 2. Hubei Provincial Engineering Research Center of Racing Horse Detection and Application Transformation，Wuhan 430056，China; 3. 华中科技大学同济医学院附属武汉儿童医院药学部，湖北 武汉 430016; 3. Department of Pharmacy，Wuhan Children’s Hospital，Tongji Medical College，Huazhong University of Science & Technology，Wuhan 430016，China

**Keywords:** QuEChERS, 超高效液相色谱-串联质谱, 非甾体抗炎药, 马尿, 残留, QuEChERS, ultra performance liquid chromatography-tandem mass spectrometry （UPLC-MS/MS）, non-steroidal anti-inflammatory drugs （NSAIDs）, horse urine, residues

## Abstract

为有效监测马匹尿液中多种非甾体抗炎药的残留，为维护马术（赛马）运动的公平性和纯洁性提供重要的技术手段，实验利用QuEChERS前处理方法结合超高效液相色谱-三重四极杆质谱技术建立了马尿中33种非甾体抗炎药的分析方法，并对QuEChERS前处理中相关参数、色谱条件和质谱条件进行了优化。具体步骤：马尿样品经乙腈提取，NaCl为盐析剂，无水MgSO_4_为除水剂，十八烷基硅烷键合硅胶（C_18_）和*N*-丙基乙二胺（PSA）为净化吸附剂，经QuEChERS净化，离心后，上清液氮吹复溶，待测分析物经Agilent Poroshell 120 EC-C_18_柱（100 mm×3.0 mm，2.7 μm）分离，以0.1%甲酸水和甲醇为流动相，梯度洗脱，正、负离子切换动态多反应监测（DMRM）模式检测，基质匹配标准曲线外标法定量。结果表明，该方法可用于马尿中33种非甾体抗炎药同时快速测定，检出限（LOD）为0.1~1.0 μg/L，定量限（LOQ）为0.9~5.4 μg/L，在0.9~220 μg/L范围内线性关系良好，相关系数均大于0.99。在LOQ、20 μg/L和200 μg/L 3个添加水平下，马尿中33种非甾体抗炎药的平均回收率为70.8%~123.8%，相对标准偏差（RSD）为1.9%~14.8%。采用本方法对10份马尿样品进行检测，共检出酮咯酸、氯诺昔康、萘普生3种禁用物质，含量均≤6.87 μg/L。酮咯酸和萘普生为受限物质，休赛期允许使用；氯诺昔康为严禁物质，不允许马匹使用。该方法简单、高效、准确，适用于马尿中33种非甾体抗炎药残留的同时测定。

## Abstract (en)

Non-steroidal anti-inflammatory drugs （NSAIDs） are often used to treat horses’ injuries and pain due to long-term training and competitions. In equestrian competitions， NSAIDs do not affect the upper limit of a horse’s athletic ability， but they can enable the horse to perform close to its maximum capacity by reducing inflammation and pain. This not only affects the competition results， but also fails to ensure the health and welfare of the horses. Therefore， it has become crucial to effectively monitor and control NSAIDs residues during equestrian competition. In this study， a rapid screening and determination method for 33 NSAIDs in horse urine was established using QuEChERS pretreatment combined with ultra performance liquid chromatography-tandem mass spectrometry （QuEChERS-UPLC-MS/MS）. The pretreatment process， chromatographic conditions， and mass spectrometric conditions were also optimized. In brief， the target analytes in 1 mL of horse urine samples were extracted with 5 mL of acetonitrile. During extraction， 100 mg of NaCl was added as a salting-out agent， and the mixture was shaken for 3 min. Subsequently， the extract was purified using a QuEChERS method with 300 mg of anhydrous MgSO_4_， 40 mg of octadecylsilane-bonded silica （C_18_）， and 40 mg of primary secondary amine （PSA） as sorbents. After centrifugation， the supernatant was dried under nitrogen and reconstituted. The 33 NSAIDs were separated on an Agilent Poroshell 120 EC-C_18_ analytical chromatographic column （100 mm×3.0 mm，2.7 μm） with gradient elution using （A） 0.1% formic acid aqueous solution and （B） methanol solution as the mobile phases. The gradient elution program was as follows： 0–1 min， 30%B； 1–3 min， 30%B–60%B； 3–12 min， 60%B–85%B； 12–12.1 min， 85%B–95%B； 12.1–14 min， 95%B； 14–14.1 min， 95%B–30%B； 14.1–17 min， 30%B. Then， the target analytes were determined by UPLC-MS/MS in dynamic multiple reaction monitoring （dMRM） mode with positive/negative ion switching. Quantification was performed using the matrix-matched external standard method. The results showed that the method can rapidly and simultaneously determine the 33 NSAIDs in horse urine. The analytes had good linear relationships within their respective ranges， with correlation coefficients of >0.99. The limits of detection （LODs） and quantification （LOQs） were 0.1–1.0 μg/L and 0.9–5.4 μg/L， respectively. The recoveries of the 33 NSAIDs in horse urine were between 70.8% and 123.8% at three spiked levels of LOQ， 20 μg/L and 200 μg/L. The relative standard deviations of all targeted compounds ranged from 1.9% to 14.8%. The method was applied to 10 actual horse urine samples. The results revealed the presence of three NSAIDs， including ketorolac， lornoxicam and naproxen， with contents ranging from < LOQ to 6.87 μg/L. According to the Equine Prohibited Substances List published by the Federation Equestre Internationale （FEI）， ketorolac and naproxen are controlled medications. They are prohibited for use during competition period， as they may mask symptoms and aggravate clinical conditions. However， they are permitted for use during non-competition times. Lornoxicam is a banned substance and is strictly prohibited for use in horses at any time. The proposed method is simple， efficient and accurate， and is suitable for the simultaneous determination of the 33 NSAIDs residues in horse urine samples.

非甾体抗炎药（non-steroidal anti-inflammatory drugs，NSAIDs）具有解热、镇痛和抗炎作用，在运动医学领域常被用于治疗运动员因长期训练及比赛造成的肌肉、骨骼和软骨组织等各种损伤和炎症^［1-8］^。NSAIDs是治疗马匹骨骼、肌肉、软组织疼痛和炎症（如急性滑膜炎、骨关节炎、跛行等疾病）的主要药物。在马术（赛马）运动中，NSAIDs不能提高马匹运动能力上限，但可通过掩盖马匹发病症状（如减轻马匹的炎症和疼痛）干扰赛前马匹跛行检查，使参赛马匹在比赛中表现得更接近其最大运动能力^［9-11］^。长期使用NSAIDs还可引起胃肠、肝肾、皮肤等系统损害，增加心血管疾病和应力性骨损伤的风险^［12-16］^。国际马术联合会（International Equestrian Federation，FEI）公布的《2025国际马联兴奋剂禁用清单》中明确规定双氯芬酸、美洛昔康、酮洛芬等非甾体抗炎药属于马匹禁用物质^［17］^。因此，建立准确、高效的检测马尿中NSAIDs残留分析方法，对维护马术（赛马）运动的公平性和纯洁性及保障马匹健康具有较大的应用价值。

我国马术运动历史悠久，但马匹禁用物质尤其是NSAIDs类违禁物质的研究和检测标准相对较少^［18-21］^，限制了我国马术产业的健康发展。开发一种准确度高、重复性好、可同时准确检测马尿中多种NSAIDs残留的分析方法，不仅是当下药物残留分析研究的热点，也可为我国马匹兴奋剂残留检测标准的建立提供基础数据^［22-24］^。Meucci等^［25］^利用HPLC-UV检测马血液中5种NSAIDs，检出限为5 ng/mL。该方法仅可检测具有紫外吸收的NSAIDs，检测药物种类范围相对较窄且灵敏度低。质谱法（MS）克服了以上两个缺点，常与气相色谱仪（GC）或液相色谱仪（LC）联用检测NSAIDs。气相色谱-串联质谱法（GC-MS/MS）检测NSAIDs时，大部分化合物需衍生化处理^［26-30］^。在样品前处理方法开发阶段，衍生试剂的用量、衍生反应温度和衍生化时间均需通过实验考察以确定最优参数。衍生化这一步骤，操作复杂且极易增加实验的不确定度。液相色谱-串联质谱法（LC-MS/MS）检测NSAIDs时，无需对待测物进行衍生化处理，主要适用于多组分已知化合物的定量分析，也可基于离子对丰度比和保留时间进行定性分析，在兴奋剂残留检测中应用较广^［31-35］^。QuEChERS方法是在分散固相萃取技术基础上发展起来的一种新型样品前处理方法，首先利用有机溶剂提取目标分析物，然后利用净化吸附剂与基质中的杂质相互作用，吸附杂质，从而达到除杂净化的目的^［36，37］^。目前该方法主要用于食品农药残留及环境监测等方面，近年来开始应用于兽药（如磺胺类、喹诺酮类、激素类等药物）残留检测领域。虽然QuEChERS方法较为成熟，但鲜少将其系统地优化并应用于马尿中多达30余种NSAIDs禁用物质的同时检测^［38-44］^。

本研究采用单因素变量和正交试验考察了QuEChERS前处理方法中提取试剂、盐、吸附剂等因素对各NSAIDs提取效果的影响，基于UPLC-MS/MS法，建立了马尿中33种NSAIDs的检测方法。该方法具有快速、灵敏、准确等特点，以期为我国马匹兴奋剂检测标准的建立提供研究基础，也为马术赛事的公平、公正提供技术参考。

## 1 实验部分

### 1.1 仪器、试剂与材料

1290（Ⅱ）-6475超高效液相色谱-三重四极杆质谱仪（美国Agilent公司）；Vortex-5涡旋混匀器（海门市其林贝尔仪器制造有限公司）；Centrifuge 5810 R离心机（德国Eppendorf公司）。

乙腈、甲醇（色谱级，美国Fisher公司）；甲酸（质谱级）和NaCl（分析级）购自上海阿拉丁生化科技股份有限公司；无水MgSO_4_（分析级）和十八烷基硅烷键合硅胶（C_18_，高纯级）购自上海麦克林生化科技股份有限公司；*N*-丙基乙二胺（PSA）购于杭州微米派科技有限公司。水杨酰胺标准品（纯度99.9%，德国HPC Standards公司）；苯恶洛芬（纯度98.0%，美国MCE公司）；依尔替酸（纯度98.0%，美国CATO公司）。酮咯酸（纯度98.5%）、吲哚布洛芬（纯度99.7%）、氟芬那酸（纯度99.5%）均购自英国LGC公司。其余27种标准品（纯度≥97.2%）均购自江苏坛墨质检科技股份有限公司。

马尿样品来自武汉商学院体育学院·国际马术学院。

### 1.2 标准溶液的配制

精密称取33种NSAIDs标准品适量，用甲醇溶解稀释，制成1 mg/mL的单一标准物质储备液，-18 ℃以下避光保存。分别精密移取33种NSAIDs的单一标准物质储备溶液10 μL，置于100 mL棕色容量瓶中，用甲醇稀释定容，配制成100 μg/L的标准溶液。

### 1.3 样品前处理

将采集的马尿样品置于50 mL塑料离心管中，置于-80 ℃超低温冰箱保存。

精密移取解冻至室温的马尿1 mL于10 mL离心管中，加入5 mL乙腈、100 mg NaCl，加盖涡旋3 min后加入300 mg无水MgSO_4_、40 mg C_18_、40 mg PSA，涡旋3 min，以4 000 r/min的转速离心10 min。取上清液至试管中，40 ℃水浴中氮吹至干后，用1 mL甲醇复溶，过0.22 μm有机相滤膜，供UPLC-MS/MS分析。

### 1.4 UPLC-MS/MS条件

#### 1.4.1 色谱条件

色谱柱为Agilent Poroshell 120 EC-C18柱（100 mm×3.0 mm，2.7 μm）；流动相：A相为0.1%甲酸水溶液，B相为甲醇。梯度洗脱程序：0~1 min，30%B；1~3 min，30%B~60%B；3~12 min，60%B~85%B；12~12.1 min，85%B~95%B；12.1~14 min，95%B；14~14.1 min，95%B~30%B；14.1~17 min，30%B。流速：0.4 mL/min；柱温：35 ℃；进样量：2 μL。

#### 1.4.2 质谱条件

离子源：电喷雾电离源（ESI），正、负离子切换模式；鞘气温度：350 ℃；鞘气流量：11.0 L/min；雾化气压力：241.32 kPa；干燥气温度：300 ℃；干燥气流量：8 L/min；毛细管电压：4 000 Ｖ。动态多反应监测（DMRM）模式，其他参数见表1。

**表1:** 33种非甾体抗炎药的保留时间、母离子、子离子、碎裂电压、碰撞能量

Compound	*t*_R_/min	Parent ions （*m/z*）	Product ions （*m/z*）	Fragmentors/V	Collision energies/eV
Fenspiride （芬司匹利）	1.94	261.2	105.1^*^， 77.1	127	30， 60
Cinchophen （辛可芬）	6.47	250.1	128.0^*^， 116.0	150	40， 40
Piroxicam （吡罗昔康）	5.19	332.1	95.1^*^， 78.1	110	18， 60
Niflumic acid （尼氟灭酸）	9.63	283.1	265.1^*^， 245.1	150	20， 40
Propyphenazone （异丙安替比林）	5.19	231.1	56.0^*^， 189.1	150	40， 20
Meclofenamic acid （甲氯灭酸）	10.77	296.0	278.1， 243.1^*^	90	11， 30
Salicylamide （水杨酰胺）	3.30	138.0	121.1^*^， 65.0	100	15， 30
Apazone （阿扎丙宗）	4.65	301.2	189.2^*^， 145.2	110	26， 56
Ketorolac （酮咯酸）	5.75	256.1	105.1^*^， 77.1	120	19， 52
Benoxaprofen （苯恶洛芬）	10.36	302.1	256.1^*^， 270.0	150	40， 40
Ketoprofen （酮洛芬）	6.47	255.1	77.1， 209.2^*^	120	52， 11
Tiaprofenic acid （噻洛芬酸）	6.16	261.1	105.1^*^， 77.0	150	20， 40
Fenbufen （芬布芬）	7.18	255.1	237.1， 181.2^*^	90	7， 26
Acemetacin （阿西美辛）	9.38	416.1	139.1^*^， 111.1	120	26， 60
Indomethacin （吲哚美辛）	9.48	358.1	139.1^*^， 111.1	110	19， 56
Indoibuprofen （吲哚布洛芬）	5.76	282.1	236.1^*^， 77.1	130	23， 60
Tolfenamic acid （托灭酸）	12.06	262.1	244.1^*^， 209.2	100	15， 30
Diclofenac （双氯芬酸）	9.35	296.0	214.1^*^， 215.1	90	38， 19
Rofecoxib （洛非考昔）	4.50	315.1	269.1， 189.1^*^	120	23， 60
Celecoxib （塞来昔布）	8.68	382.1	362.1^*^， 282.1	150	20， 40
Etoricoxib （依托考昔）	4.47	359.1	280.1^*^， 279.1	150	40， 40
Oxaprozin （奥沙普嗪）	8.70	294.1	103.2^*^， 276.2	120	34， 15
Etodolac （依托度酸）	9.19	288.2	172.2^*^， 143.2	80	11， 45
Carprofen （卡洛芬）	9.23	274.1	228.1， 193.0^*^	110	15， 35
Mefenamic acid （甲灭酸）	11.27	242.1	224.2^*^， 209.2	100	15， 34
Meloxicam （美洛昔康）	6.33	352.0	115.1^*^， 73.0	110	19， 60
Deracoxib （地拉考昔）	6.24	398.1	378.1^*^， 283.1	140	30， 41
Suprofen （舒洛芬）	5.73	261.1	111.1^*^， 177.2	100	22， 19
Eltenac （依尔替酸）	8.75	302.0	221.1^*^， 220.1	100	23， 41
Antipyrine （安替比林）	3.20	189.1	56.0^*^， 77.1	130	37， 45
Lornoxicam （氯诺昔康）	5.37	372.0	121.1， 78.1^*^	123	22， 60
Naproxen （萘普生）	6.96	231.1	185.2^*^， 170.2	97	15， 30
Flufenamic acid （氟芬那酸）	11.08	282.1， 280.1	264.1， 236.2^*^	100， 110	11， 19

* Quantitative ion.

### 1.5 数据库的建立

本实验将1.2节的33种NSAIDs标准溶液按上述仪器分析条件进样，获得该条件下的保留时间、母离子、子离子、碎裂电压和碰撞能量，利用MassHunter软件建立33种NSAIDs数据库。

## 2 结果与讨论

为提高前处理工作效率，本研究采用QuEChERS法进行提取、净化。尿液基质主要成分是水分，此外还含有蛋白质、葡萄糖、尿素以及无机盐等内源性物质，这些内源性物质会对残留的NSAIDs目标物的分析造成干扰，也会对质谱仪造成污染，需要有效提取及净化方法去除杂质干扰。本实验采取单因素试验法考察不同前处理因素对各目标物提取效果的影响，用绝对回收率（加标样品的峰面积/标准溶液的峰面积×100%）表示提取效果。

### 2.1 提取溶剂的选择

实验分别考察了不同提取溶剂（乙腈、1%甲酸乙腈、乙酸乙酯、1%甲酸乙酸乙酯、乙酸乙酯-乙腈（1∶3， *V*/*V*）、乙酸乙酯-乙腈（3∶1， *V*/*V*）、乙酸乙酯-乙腈-二氯甲烷（1∶2∶1， *V*/*V*/*V*））对33种NSAIDs的提取效果（见图1a）。

**图1:**
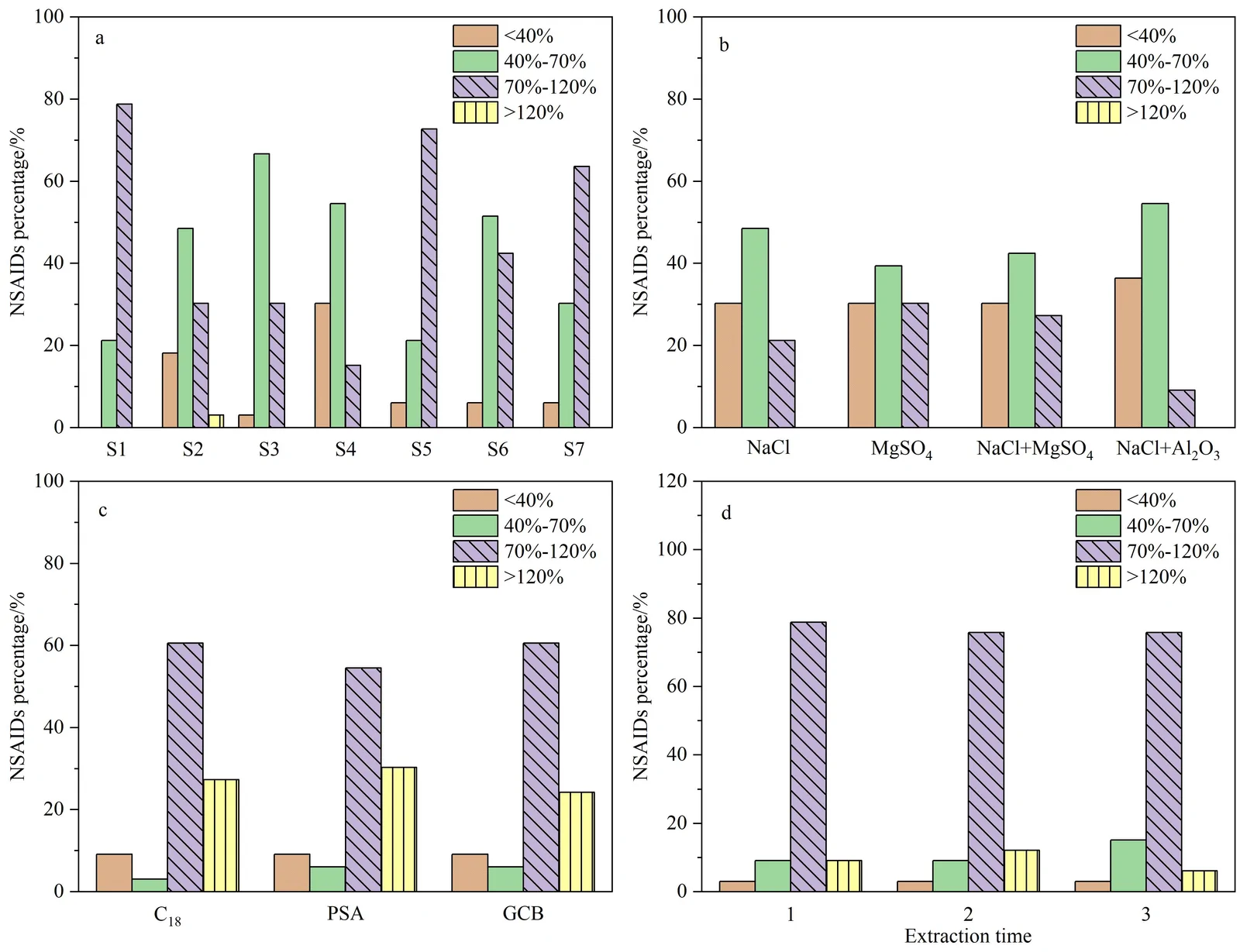
（a）提取溶剂、（b）盐、（c）吸附剂和（d）提取次数对33种NSAIDs提取效果的影响（*n*=3）

由图1a可知，乙腈作为提取溶剂时，有80%目标物（26个）绝对回收率在70%~120%范围内；乙酸乙酯-乙腈（1∶3， *V*/*V*）作为提取溶剂时，仅有72.7%目标物（24个）绝对回收率在70%~120%范围内。其中，异丙安替比林和水杨酰胺在以上两种溶剂作为提取溶剂时回收率具有明显的差异。异丙安替比林在乙腈、乙酸乙酯-乙腈（1∶3， *V/V*）作为提取溶剂时回收率分别为71.9%和47.4%，水杨酰胺在乙腈、乙酸乙酯-乙腈（1∶3， *V/V*）作为提取溶剂时回收率分别为48.5%和20.1%。此外，乙腈具有沉淀蛋白质作用且对尿液中脂质成分溶解度低，有利于在提取多种NSAIDs的同时减少内源性基质干扰，故综合考虑之下选择乙腈作为提取溶剂。

### 2.2 净化方式的选择

QuEChERS前处理中，盐可调节溶液的极性，影响提取物分配，有利于排除有机层中的极性化合物（如尿素、盐、葡萄糖等），减少基质效应的干扰。本研究考察了不同盐，包括NaCl、无水MgSO_4_、NaCl+无水MgSO_4_、NaCl+中性Al_2_O_3_对各目标物提取效果的影响。由图1b可知，目标物在无水MgSO_4_、NaCl+无水MgSO_4_ 2种提取条件下，绝对回收率介于70%~120%范围内的占比较大。因马尿样品加入提取溶剂（乙腈）后两溶液互溶，NaCl可使水相（马尿）和有机相（提取溶剂）分层，而无水MgSO_4_与水反应可吸收溶液中的水分且产生热量，有助于目标物的萃取。为更好地去除马尿基质中的杂质干扰，故选择NaCl、无水MgSO_4_作为盐析剂和除水剂。

目前，QuEChERS方法中最常用的吸附剂是PSA、C_18_、石墨化炭黑（GCB）。PSA为弱离子交换剂，可有效去除脂肪酸、色素和糖类等，C_18_多用于吸附油脂和蛋白质等非极性物质，GCB主要用于吸附色素，但对具有平面结构的物质有极强的吸附作用^［45-47］^。结果可知（图1c），C_18_、PSA和GCB对33种目标物的绝对回收率（4个范围占比）差异较小，但GCB对苯恶洛芬、卡洛芬、阿西美辛和吲哚美辛有明显的吸附作用。根据马尿基质的特点和净化除杂去干扰的目的，本研究选取C_18_和PSA联用作为吸附剂。

此外本实验考察了提取次数（1、2、3次）对33种NSAIDs提取效果的影响。由图1d可知，在提取1次的条件下，绝对回收率介于70%~120%的目标物数量较提取2次及3次条件下占比更高。具体对比不同提取次数中各目标物回收率发现，提取次数对目标物的提取效果影响较小，因此最终选择提取1次。

### 2.3 正交试验优化QuEChERS前处理参数

本研究以33种NSAIDs的绝对回收率为评价指标，采用L_9_（3^4^）正交设计考察A（乙腈）、B（无水MgSO_4_）、C（C_18_）和D（PSA）的加入量对结果的影响（见表2）。以33种NSAIDs绝对回收率的平均值作为综合得分，利用*K*和*R*值分析33种目标物的最优条件（见表3）。*K*值表示化合物在每种条件下的绝对回收率总和。*R*值是因素中最高水平和最低水平绝对回收率的差值。*R*值越大，表明该因素对马尿样品前处理效果的影响越显著^［37，48］^。

**表2:** 正交试验因素水平

Level	A（Acetonitrile/mL）	B（Anhydrous MgSO_4_/mg）	C（C_18_/mg）	D（PSA/mg）
1	3	300	20	30
2	4	400	30	40
3	5	500	40	50

**表3:** 正交试验结果及极差分析结果

No.	Columns				Averagerecovery/%
	A	B	C	D	
1	1	1	1	1	73.2
2	1	2	2	2	77.1
3	1	3	3	3	75.4
4	2	1	2	3	77.0
5	2	2	3	1	76.8
6	2	3	1	2	76.9
7	3	1	3	2	81.0
8	3	2	1	3	77.0
9	3	3	2	1	78.3
*K*_1_	225.6	231.3	227.1	228.3	
*K*_2_	230.7	231.0	232.5	234.9	
*K*_3_	236.4	230.7	233.4	229.5	
*k*_1_	75.2	77.1	75.7	76.1	
*k*_2_	76.9	77.0	77.5	78.3	
*k*_3_	78.8	76.9	77.8	76.5	
*R*	3.6	0.2	2.1	2.2	
Order	A>D>C>B				
Optimal level	A_3_	B_1_	C_3_	D_2_	

直观分析：由极差*R*可以看出，对综合评价影响大小依次为A>D>C>B；对A因素影响为*K*_3_>*K*_2_>*K*_1_；对B因素影响为*K*_1_>*K*_2_>*K*_3_；对C因素影响为*K*_3_>*K*_2_>*K*_1_；对D因素影响为*K*_2_>*K*_3_>*K*_1_。其中，A因素中有27个NSAIDs最优在3水平；B因素中有12个NSAIDs最优在1水平；C因素中有20个NSAIDs最优在3水平；D因素中有29个NSAIDs最优在2水平。综合以上分析，对马尿前处理结果影响最大的因素是乙腈加入量，其次为因素PSA加入量，最小影响因素是无水MgSO_4_加入量，前处理过程最优参数为A_3_B_1_C_3_D_2_。即用5 mL乙腈提取后，加入300 mg 无水MgSO_4_除水，40 mg C_18_和40 mg PSA 吸附净化除杂。

### 2.4 色谱-质谱条件优化

本实验选用Agilent Poroshell 120 EC-C_18_作为分离柱，有机相为甲醇，水相为0.1%甲酸水。在流动相中加入0.1%甲酸能够显著提高各目标物的响应值。通过优化，采用1.4节梯度洗脱条件，各化合物的保留时间见表1。

分别采用正离子扫描模式（ESI^+^）和负离子扫描模式（ESI^‒^）对33种NSAIDs标准溶液进行母离子扫描，除氟芬那酸在正离子和负离子扫描模式下均能得到准离子峰外，其余目标物采用正离子扫描模式均可产生稳定的［M+H］^+^准分子离子峰。对33种NSAIDs的子离子、碎裂电压、碰撞能量等参数进行优化，优化后的质谱分析参数详见表1。利用MassHunter软件建立33种NSAIDs数据库，利用数据信息建立DMRM方法。

### 2.5 基质效应的评价

基质效应是指样品中除了目标分析物以外的其他成分对目标物检测结果的影响。本研究分别用溶剂和空白马尿基质溶液配制1、5、10、20、50、100、200 μg/L混合标准溶液，以峰面积对相应的质量浓度做标准曲线，比较两条标准曲线斜率，判断基质效应的强弱^［49］^。计算公式：基质效应=（基质匹配标准曲线的斜率/溶剂标准曲线的斜率-1）×100%。结果如表4所示，28种NSAIDs的基质效应为基质抑制效应，其中10个NSAIDs基质效应的绝对值大于20%，即基质抑制效应显著；5种NSAIDs为基质增强效应，其中2个NSAIDs的基质效应大于20%，即基质增强效应显著。马尿基质复杂，含有大量尿素、盐、肌酐、激素代谢物、葡萄糖、色素及各种外源性物质。PSA（主要用于去除脂肪酸、色素和糖类等）和C_18_（主要用于吸附油脂和蛋白质等非极性物质）组合，虽然能有效去除部分极性和非极性干扰物，但对于某些与目标物性质非常接近的共萃取物或离子型化合物的去除能力可能确实有限，这导致了分析中出现离子抑制或增强效应基质效应显著的现象。为确保检测结果的准确性和重复性，本研究通过使用空白马尿基质配制标准曲线定量分析，尽可能减小基质效应带来的定量偏差。

**表4:** 马尿中33种非甾体抗炎药的决定系数、回收率、相对标准偏差、检出限、定量限及基质效应（*n*=6）

Compound	*R*^2^	LOD/ （μg/L）	LOQ/ （μg/L）	LOQ		20 μg/L		200 μg/L		ME/%
				Recovery/%	RSD/%	Recovery/%	RSD/%	Recovery/%	RSD/%	
Fenspiride	0.9995	0.1	1.1	75.2	7.2	103.5	6.7	92.8	5.9	-84.9
Cinchophen	0.9997	0.1	1.1	96.5	4.3	97.7	7.4	95.6	4.6	-31.8
Piroxicam	0.9983	0.1	1.0	78.2	7.5	99.2	6.8	91.3	5.6	-12.4
Niflumic acid	0.9997	0.5	1.0	98.2	4.9	99.2	7.1	95.6	5.5	-14.1
Propyphenazone	0.9995	0.1	1.1	85.4	7.2	92.3	8.0	88.3	7.6	-13.9
Meclofenamic acid	0.9998	0.5	0.9	76.5	11.7	102.4	6.1	99.0	5.3	-10.7
Salicylamide	0.9994	0.1	5.4	75.3	7.1	87.2	5.4	89.2	5.0	-26.9
Apazone	0.9976	0.1	1.0	71.0	4.5	78.4	7.33	75.9	3.3	14.4
Ketorolac	0.9998	0.1	1.0	107.7	3.2	103.6	8.0	93.8	5.2	-25.2
Benoxaprofen	0.9997	0.1	0.9	91.6	9.9	98.0	6.2	96.6	5.1	-10.6
Ketoprofen	0.9986	0.5	1.1	93.3	13.3	95.4	9.5	92.0	3.4	-5.8
Tiaprofenic Acid	0.9995	0.1	1.0	118.3	6.5	100.2	7.8	90.6	5.6	-14.8
Fenbufen	0.9999	0.5	1.0	116.6	8.8	99.6	8.6	95.2	6.1	-12.7
Acemetacin	0.9994	0.5	1.0	123.8	4.4	100.0	7.2	100.3	6.0	4.77
Indomethacin	0.9997	0.1	4.8	78.3	11.3	102.9	9.3	94.5	5.3	26.9
Indoibuprofen	0.9999	0.5	1.0	93.4	14.8	104.8	6.8	92.4	7.0	31.6
Tolfenamic acid	0.9996	0.5	1.0	76.8	12.9	106.6	7.0	103.1	5.9	-13.3
Diclofenac	0.9998	0.1	1.1	105.3	5.7	101.5	8.1	95.0	3.7	-10.6
Rofecoxib	0.9987	1.0	5.3	80.2	7.1	94.1	10.4	83.1	4.6	-71.6
Celecoxib	0.9999	1.0	5.4	91.6	11.0	101.8	8.6	92.8	7.2	-36.6
Etoricoxib	0.9988	0.1	0.9	101.4	9.5	95.0	8.2	95.5	3.7	-16.7
Oxaprozin	0.9999	0.1	1.0	101.4	4.7	98.1	7.0	94.4	6.5	-5.8
Etodolac	0.9995	0.1	1.1	92.5	5.8	91.7	7.5	91.4	1.9	-2.2
Carprofen	0.9996	0.5	1.1	95.5	14.1	101.4	6.3	92.7	5.9	-14.7
Mefenamic acid	0.9998	0.1	1.1	95.4	10.7	102.5	7.0	101.4	5.9	-17.2
Meloxicam	0.9999	0.1	1.0	116.3	3.7	93.9	6.6	92.0	4.9	-10.4
Deracoxib	0.9997	0.1	0.9	73.1	8.0	97.4	8.6	91.5	4.8	-42.8
Suprofen	0.9999	0.5	1.1	119.4	9.9	99.4	5.7	91.9	5.3	-27.1
Eltenac	0.9997	0.5	1.0	102.6	7.1	98.0	7.6	96.3	4.4	-9.47
Antipyrine	0.9990	0.1	1.0	93.5	5.1	104.3	6.8	96.6	5.0	-20.5
Lornoxicam	0.9998	0.5	5.2	117.4	5.2	104.6	7.8	93.8	5.6	-13.4
Naproxen	0.9997	0.5	1.1	70.8	9.4	98.8	7.1	93.7	5.1	-29.0
Flufenamic acid	0.9965	0.5	5.2	89.8	4.2	93.7	7.2	83.8	6.2	15.7

### 2.6 方法学考察

用空白马尿基质配制不同系列浓度的标准溶液，在1.4节分析条件下进行测定，以目标物的定量离子峰面积（*y*）对质量浓度（*x*，μg/L）作标准曲线，33种NSAIDs的决定系数（*R*^2^）均大于0.99（见表4），表明其线性关系均良好。在空白马尿中加标，按照1.3节方法前处理后分析检测，信噪比（*S*/*N*）以不小于3确定检出限（LOD），为0.1~1.0 μg/L；信噪比以不小于10确定定量限（LOQ），为0.9~5.4 μg/L，各目标物在定量限水平的加标回收率为70.8%~123.8%，RSD（*n*=6）均小于15%，说明以上定量限可用于准确定量。

在空白马尿样品中添加33种NSAIDs的混合标准溶液，添加水平分别为LOQ、20 μg/L和200 μg/L，按照1.3节和1.4节方法进行提取，净化和检测。每个水平重复6次，33种目标物的平均回收率为70.8%~123.8%，相对标准偏差为1.9%~14.8%（见表4）。

### 2.7 与其他方法的比较

本研究与已报道的检测尿液、血液样本中非甾体抗炎药的相关研究进行对比，所开发的方法前处理通量远高于其他方法，且操作简单，灵敏度高，成本低，实用性强（见表5）。

**表5:** 本方法与其他方法的比较

Sample pretreatment method	Matrix	Detection	Number of compounds	Linear range/ （μg/L）	LOQ/（μg/L）	Ref.
LLE	equine plasma	LC-MS	16	5.0-200	1.0-5.0	［^50^］
SPE	equine urine	GC-MS	9	25-375	53.0-267.2	［^26^］
MSB-LPME	human serum	LC-MS	4	-	0.83-3.16	［^51^］
SPMTE	human urine	LC-UV	4	10-10000	11-31	［^52^］
μSPE	whole blood	LC-UV	3	50-2000	50-75	［^53^］
MISPE	equine plasma	LC-UV	7	80-250000	10-80	［^54^］
QuEChERS	equine urine	LC-MS	33	0.9-220	0.9-5.4	this study

LLE： liquid-liquid extraction； SPE： solid phase extraction； MSB-LPME： magnetic solvent bar liquid-phase microextraction； SPMTE： solid phase membrane tip extraction； μSPE： micro solid phase extraction； MISPE： molecular imprinted solid phase extraction； UV： ultraviolet.

### 2.8 实际样品的检测

应用本研究所建立的方法对10份温血马的马尿样品进行检测，将检出的色谱峰保留时间、离子丰度比分别与标准品色谱峰的保留时间、离子丰度比相比较，经确证，共检出3种化合物，保留时间偏差均在±0.1 min之内，检测结果见表6，代表性MRM图谱如图2所示。

**表6:** 10份马尿样品的检测结果（*n*=3）

Sample No.	Place of birth	NSAID residues/（μg/L）		
		Ketorolac	Lornoxicam	Naproxen
1	Belgium	1.05	<LOQ	6.87
2	Holland	1.10	<LOQ	-
3	Belgium	<LOQ	<LOQ	-
4	Belgium	<LOQ	<LOQ	-
5	Germany	1.01	<LOQ	-
6	Holland	-	<LOQ	-
7	Belgium	-	-	<LOQ
8	Belgium	<LOQ	<LOQ	-
9	Belgium	-	<LOQ	-
10	Belgium	-	<LOQ	-

**图2:**
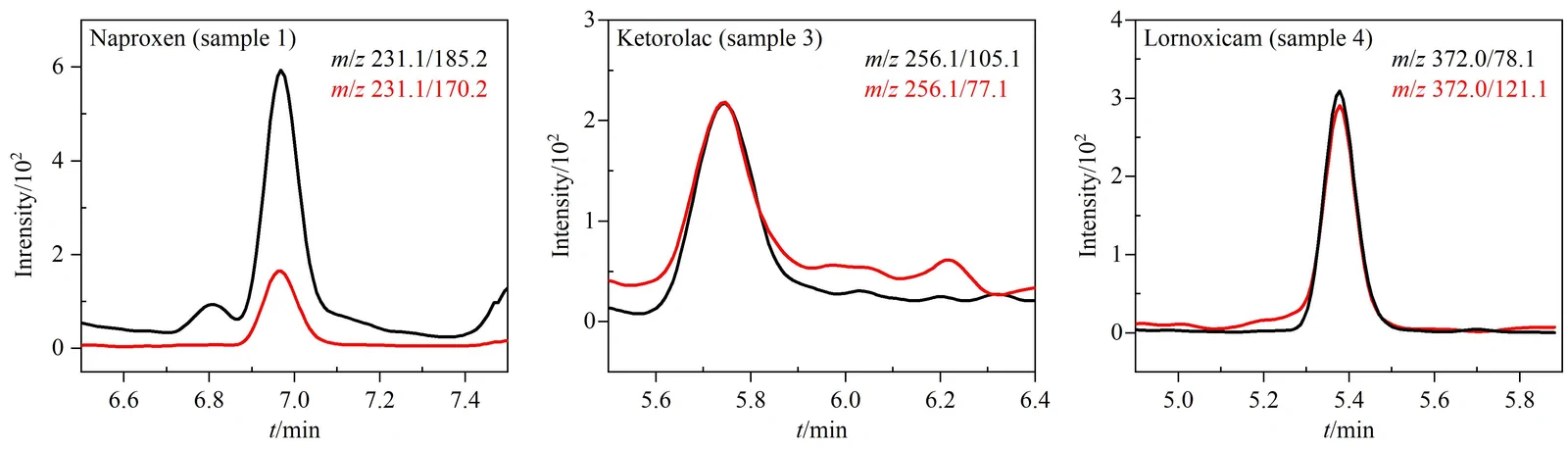
马尿样品中3种非甾体抗炎药的代表性MRM色谱图

10份马尿中有6份马尿检出酮咯酸，其中3份含量小于定量限，其余尿液的药物含量分布在1.01~1.10 μg/L。9份马尿检出氯诺昔康，含量均小于定量限。2份马尿样品检出萘普生，1份含量低于定量限，另一份含量为6.87 μg/L。根据FEI发布的马匹兴奋剂清单中可知，酮咯酸和萘普生为受限物质（受限物质为在特定时间内（一般是赛内），不允许对马匹使用，不允许在马匹体内检测到的物质或有相同或类似生物作用的物质）。因为在比赛期使用，可能会掩盖症状，加重病情。10份样品是在无赛事期间采集的，酮咯酸和萘普生用于马匹的治疗是合理的。氯诺昔康为严禁物质（严禁物质为不允许对马匹使用、尝试使用、不允许在马匹体内检测到的物质或有相同或类似生物作用的物质）。在9份马尿中均有检出，虽低于定量限，但需引起重视。

## 3 结论

本文以马尿为研究基质，利用QuEChERS前处理技术结合UPLC-MS/MS，建立了检测马尿中33种非甾体抗炎药残留量的方法。结果表明，该方法可同时对马尿中33种NSAIDs进行快速筛查及准确测定，具有前处理通量高、效率高、成本低、灵敏度高、对马尿样品专属性高、实用等特点，可为我国马匹反兴奋剂检测标准的制定提供方法参考和基础数据。
